# Corrosion inhibition of mild steel in hydrochloric acid solution by the expired Ampicillin drug

**DOI:** 10.1038/s41598-023-33519-y

**Published:** 2023-04-25

**Authors:** Khalid A. Alamry, Ajahar Khan, Jeenat Aslam, Mahmoud A. Hussein, Ruby Aslam

**Affiliations:** 1 Chemistry Department, Faculty of Science, King Abdulaziz University, Jeddah, 21589 Saudi Arabia; 2 Department of Food and Nutrition, Bionanocomposite Research Center, Kyung Hee University, 26 Kyungheedae-ro, Dongdaemun-gu, Seoul, 02447 South Korea; 3 Department of Chemistry, College of Science, Taibah University, Yanbu-30799, Al-Madina Saudi Arabia; 4 Corrosion Research Laboratory, Department of Applied Chemistry, Faculty of Engineering and Technology, Aligarh Muslim University, Aligarh, 202002 India

**Keywords:** Chemistry, Materials science

## Abstract

This study examines the utilization of the expired drug, namely ampicillin, as a mild steel corrosion inhibitor in an acidic environment. The inhibitor was evaluated using weight loss and electrochemical measurement accompanied with surface analytical techniques. The drug showed a potential inhibitory efficiency of > 95% at 55 °C. The inclusion of the inhibitor increased the charge transfer resistance at the steel-solution interface, according to impedance analyses. According to potentiodynamic polarisation measurements, expired ampicillin drug significantly decreased the corrosion current density and worked as a mixed-type corrosion inhibitor. The Langmuir adsorption isotherm was followed by the adsorption of ampicillin drug on the steel substrate, exhibiting an association of physical and chemical adsorption mechanisms. The surface study performed using contact angle and scanning electron microscopy–energy dispersive spectroscopy (SEM–EDS) measurements supported the inhibitor adsorption on the steel substrate.

## Introduction

Mild Steel (MS) is usually employed in petroleum production, chemical processing, construction engineering, marine, automobile, aerospace, etc.^1–3^. When mild steel comes into contact with corrosive media during some industrial processes, such as pickling, acid descaling, boiler cleaning, oil well acidification, and acid fracturing, the mild steel becomes severely corroded^4,5^. Corrosion is a process that occurs when a metal's surface gets damaged due to its interaction with environment. It can be caused by the exposure of the metal to a liquid or gas. It can lead to a financial loss due to its dangerous and hazardous effects. Various methods have been presented to prevent this type of damage. Some of these include the use of corrosion inhibitor^6–11^, coatings^12–14^, modifying the damaging climate, and the use of anodic protection^15^ etc. One of the most crucial methods that can be used during the pickling process is the use of a corrosion inhibitor. Effective steel corrosion inhibitors include chromate and organic molecules. However, due to chromate’s high toxicity, Registration, Evaluation, Authorization, and Restriction of Chemicals (REACH) regulations prohibit its practical application^16,17^. Organic molecules are limited by poor solubility, poor volatility, and poor biodegradability^18^. Since most corrosion inhibitors exhibit toxic behaviour, it is possible to adhere to environmental sustainability and safety principles by using natural inhibitors, such as plant extracts, bio-polymers, amino acids and drugs etc.

Numerous reports have shown that the employment of pharmaceuticals in treating corrosion is friendly approach and does not have a negative effect on the environment^10^. Researchers claim that drugs can contest with green corrosion inhibitors. Majority of them can be produced using natural resources. Carbocyclic or heterocyclic systems are very common in drug structures. Various factors affect the selection of the appropriate drug for treating corrosion. First, the molecules used to treat corrosion must contain nitrogen, oxygen, and sulphur. Second, the drug must be environmentally friendly since it can catalyze biological reactions. Large molecular size and solubility are essential factors that can help determine the ideal drug for corrosion treatment. As per the literature survey, many types of drugs such as Tramadol, Cephapirin, Tenormin^19^, Phenobarbital^20^, Ethambutol^21^, Cephapirin^2^, spironolactone^22^, Atenolol^23^, Cephalothin^24^, Telmisartan^25^, Modiaquine^26^, Ciprofloxacin^27^, Glimepiride^28^, Ibuprofen and Diclofenac^29^, Salazopyrin^30^, Tramadol^31^, Cefixime and Cefpirome^32^, Moxifloxacin and Betamethasone^33^, Amoxicillin, Ciprofloxacin, Doxycycline, and Streptomycin^34^, Tetracycline and Streptomycin^35^ etc. have been effectively employed as sustainable inhibitors for the control of corrosion of different metals and alloys. However, most of these are expensive compared to the organic inhibitors in use^36^. Although it's believed that most drugs can retain their inventive efficacy after expiration, however, their practice for other purposes is restricted because of their liability and professional limitations^37^. The excessive use of medicine can lead to the accumulation of toxic substances in the environment and homes. It can also affect wildlife by causing toxic effects^38^. According to the estimates, the cost of drug wastage in Saudi Arabia is about $150 million annually^39^. The average drug wastage was 25.8% in Saudi Arabia, and in other Gulf regions, the wastage rate is around 41.3%^40^. Therefore, this is not only a critical issue in Saudi Arabia but also has a global impact in both developed and developing countries^36^.

Currently research works were conducted using expired drugs as corrosion inhibitors. Expired drugs or unused drugs in the environment results in negative impact on human, aquatics as well as terrestrial eco-system thereby the use of expired drugs as corrosion inhibitors leads to the effective waste management of expired drugs and also reduces the economic losses^36^. Many expired drugs such as carbamazepine and paracetamol^36^, Atenolol and Nifedipine^37^, declophen(2-(2,6-dichloranilino) phenylacetic acid) ampoules^41^, Voltaren^42^, Asthalin^43^, Atorvastatin^44^, Carvedilol^45^ for carbon steel, ampicillin (AMP) and ceftriaxone (CRO) for Sabic Fe corrosion^46^, Meloxicam^47^, Levothyroxine for SS^48^, Oseltamivir for Aluminium^49^, etc. were reported as sustainable inhibitors.

As a result, in the current study, we chose an expired antibiotic drug namely ampicillin. Ampicillin is a semi-synthetic penicillin derivative that acts as an orally active broad-spectrum antibiotic. It treats and prevents various bacterial infections, including urinary tract infections, respiratory tract infections, meningitis, endocarditis and salmonellosis. It may also be used to protect new-borns from group B streptococcal infection. It's application to inhibit mild steel corrosion in 5% HCl at different temperatures was studied. The corrosion studies include weight loss measurement, potentiodynamic polarization measurement, electrochemical impedance, and contact angle measurement and scanning electron microscopy–energy dispersive spectroscopy (SEM–EDS) studies.

## Experimental details

### Preparation of studied surface and tested media

**Figure 1:**
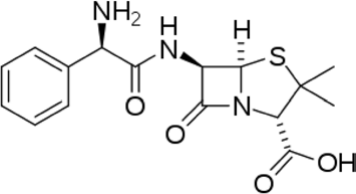
Chemical structures of the tested inhibitor.

Expired drug collected from pharmaceutical drug store. The drug was obtained in pure form and used for this study without any further purification. Figure 1 shows the structural formula of the drug employed. The composition (wt%) of mild steel used for experiments is (wt%) C, 0.061; Mn, 0.181; P, 0.017; Cr, 0.035; Al, 0.017 and remain Fe. For the weight loss measurement measurements, the MS specimens were sized into rectangular pieces that were 2.5 × 2 × 0.1 cm^3^ in size. They are then cleaned and polished using acetone and SiC paper of grades 180–1200. The specimens are then subjected to 5% HCl solution that is made by the dilution of 37% HCl in bi-distilled water. The solution was standardized using already prepared standard 1 M Na_2_CO_3_ solution.

### Weight loss measurements

The corrosion protection effect of the drug on mild steel in 5% HCl solution at varying temperatures was analysed through the weight loss method^50^.

The testing samples were suspended in 5% HCl solution in the absence and presence of various inhibitor concentrations for 6 h at 25, 35, 45 and 55 °C, respectively. Specimens were washed with water immediately after immersion and then rubbed with a soft brush; finally, the samples were washed with acetone and anhydrous ethanol and weighed accurately again after drying. In this study, the average corrosion rate (ν in mpy) and inhibition efficiency (%*η*_w_) of mild steel were calculated according to the following formulas^51^:

1 $$\nu =\frac{KW}{At\rho }$$

where *ν*—corrosion rate, *K*—constant (3.46 × 10^6^), *t—*immersion time in h, *W*—weight loss in grams, *A*—coupon’s exposed area in cm^2^, *ρ*—density of MS, whose value is 7.86 g cm^−3^.

Surface coverage (*θ*) and corrosion inhibition efficiencies (%*η*_w_) were determined through corrosion rate data by Eqs. (2) and (3), respectively.

2 $$\theta =\frac{{\nu }_{0}-{\nu }_{i}}{{\nu }_{0}}$$

3 $$\%{\eta }_{w}=\frac{{\nu }_{0}-{\nu }_{i}}{{\nu }_{0}}\times 100$$

where corrosion rates are indicated by ν_0_ and ν_i_ in the absence and presence of inhibitor, respectively.

### Electrochemical analyses

Potentiodynamic polarization (PDP) and Electrochemical impedance (EIS) measurements investigations were done using the three-electrode cell system comprising of a circular MS specimen with exposed area of 1 cm^2^ as a working electrode, Ag/AgCl saturated by 3 M KCl and platinum rod (The surface area is approximatively 1 cm^2^) as a reference and counter electrodes, respectively. Before the measurement, the MS samples were immersed in a corrosive environment for 30 min to achieve a steady-state open circuit potential (OCP) to check that the surface of working electrode attained a steady-state potential. The test was performed at a frequency range of 10^–2^ to 10^5^ Hz with a potential perturbation of 10-mV. All the experiments were performed in this investigation at 25, 35, 45 and 55 °C.

The inhibition efficacies of the inhibitor using EIS parameter i.e., polarization resistance (*R*_p_) was denoted as %*η*_E_ and calculated using Eq. (4):

4 $${\%\eta }_{E}=\frac{{R}_{p}^{(i)}-{R}_{p}^{(o)}}{{R}_{p}^{(i)}}\times 100$$

*R*^*(i)*^_p_ and *R*^o^_p_ are the polarization resistances in the presence and absence of inhibitor, respectively.

The PDP measurements evaluates the corrosion current potential and inhibition efficacy of MS that has been exposed to acidic environments both in the occurrence and absence of inhibitor within a range of + 250 to − 250 mV vs. open circuit potential (OCP) and at scan rate of 0.1 mV s^−1^. The %*η*_I_ (percent inhibition efficiency calculated by corrosion current density values) can be determined using Eq. (5).

5 $$\mathrm{\%}{\eta }_{I}=\frac{{I}_{corr}^{(o)}-{I}_{corr}^{(i)}}{{I}_{corr}^{(o)}}\times 100$$

*I*^(°)^_corr_ and *I*^(i)^_corr_ are the corrosion current densities in the absence and presence of inhibitor, respectively.

### Contact angle measurement

To determine the effective wetting characteristics of mild steel, the KRUSS Germany model FM41Mk2 Simple drop wettability analyser was used. The samples were prepared according to the weight loss measurements.

### Surface characterization methods

In order to better understand the surface film morphology and composition, the samples were investigated through SEM–EDS analysis. The specimens were analysed using the JEOL JSM-6510LV model, equipped with an INCA, Oxford energy dispersive X-ray spectrometer. Morphologies of all the coupons were recorded with a magnification of  250× to present the constant view.

## Results and discussion

### Weight loss test

The Table 1 shows the outcomes of MS corrosion behaviour in HCl with varying concentrations of expired ampicillin drug for 6 h in the temperature range of 25–55 °C. The results of this Table shows that the rate of MS corrosion and inhibition efficiencies increased with increasing temperature^52,53^. This suggests that at high temperatures, the ampicillin drug's improved performance could result from the alteration in adsorption behaviour from physical to chemical adsorption. In the case of chemical adsorption, the chemical interactions among active sites of metal and the adsorption centres of inhibitor molecules occur, making the desorption process difficult. Besides, increasing temperature results in the desorption of water molecules from the surface and increasing accessibility of the surface sites for the adsorption of inhibitor molecules. The results confirmed that the drug molecules were chemically adsorbed on the MS^54^ and are a suitable temperature-resistant corrosion inhibitor.

**Table 1:** Weight loss test results of MS following 6 h immersion in 5% HCl solution in the absence and occurrence of diverse concentrations of expired Ampicillin drug at varying temperatures.

*C* (mM)	25 °C			35 °C			45 °C			55 °C		
	*ν* (mpy)	*θ*	%*η*_*w*_	*ν* (mpy)	*θ*	%*η*_*w*_	*ν* (mpy)	*θ*	%*η*_*w*_	*ν* (mpy)	*θ*	%*η*_*w*_
0	69.12 ± 1.6	**–**	**–**	156.37 ± 5.2	**–**	**–**	526.8 ± 14.8	**–**	**–**	2342.3 ± 32.9	**–**	**–**
0.01	22.14 ± 0.3	0.679	67.9	45.63 ± 1.2	0.708	70.8	141.6 ± 4.3	0.731	73.1	82.5 ± 11.3	0.964	96.4
1	20.13 ± 0.2	0.708	70.8	40.94 ± 1.1	0.738	73.8	122.8 ± 4.1	0.766	76.6	73.1 ± 10.3	0.968	96.8
10	17.44 ± 0.2	0.747	74.7	36.24 ± 0.7	0.768	76.8	97.9 ± 2.3	0.814	81.4	72.5 ± 7.6	0.969	96.9
20	16.77 ± 0.1	0.757	75.7	34.89 ± 0.5	0.776	77.6	84.5 ± 1.1	0.839	83.9	75.1 ± 5.5	0.967	96.7

“ ± ” shows the standard deviation of three measurements.

### Assessment of Adsorption Isotherms and thermodynamic parameters

**Figure 2:**
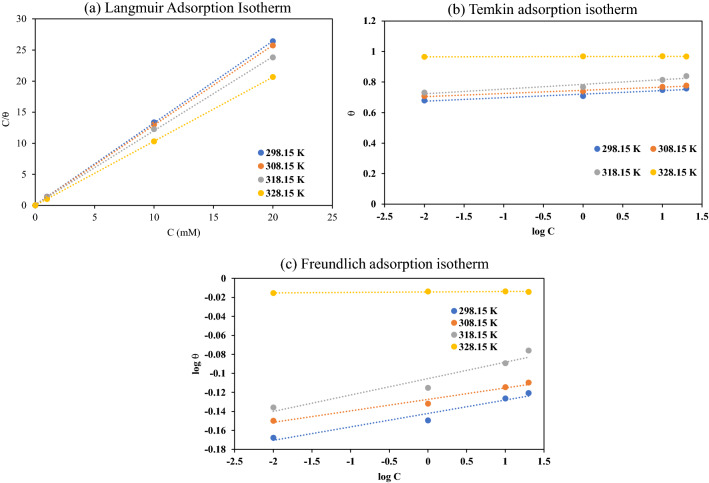
(**a**) Langmuir, (**b**) Temkin, (**c**) Freundlich adsorption isotherm.

The results of the gravimetric measurement were used to select the most appropriate isotherm for the adsorption of drug molecules on the metal surface (Fig. 2a–c). The linear regression coefficient (R^2^), meticulously related to the unity, authenticated the Langmuir adsorption model as shown in Eq. 6 where *θ* is the fraction of the surface covered by the adsorbed inhibitor molecules and *C* is the inhibitor’s concentration^55^:

6 $$\frac{C}{\theta }=\frac{1}{{K}_{ads}}+C$$

where the equilibrium adsorption constant is denoted by *K*_ads_, which can be attained as the intercept value from the plot of *C*/*θ* against *C* (Fig. 2a)_._ The conquered unit slope of Eq. (6) given in Table 2 confirmed the fitted Langmuir adsorption model. Moreover, high *K*_ads_ value at 55 °C given in Table 2 suggests the more pronounced adsorption process (Table 2).

**Table 2:** Thermodynamic characteristics of adsorption for MS in 5% HCl at different temperatures.

Temperature (°C)	Slope	*R*^*2*^			*K*_ads_	Δ*G*°_ads_ (KJ/mol)	Δ*H*°_ads_ (KJ/mol)	Δ*S*°_ads_ (J/mol/K)
		Langmuir adsorption isotherm	Temkin	Freundlich adsorption isotherm				
25	1.32E + 00	1	0.942	0.948	13.7	− 41.4	40.49	− 0.270
35	1.29E + 00	1	0.969	0.972	17.4	− 43.39	40.49	− 0.276
45	1.19E + 00	0.9998	0.984	0.924	82.5	− 48.95	40.49	− 0.295
55	1.03E + 00	1	0.730	0.730	384.6	− 54.72	40.49	− 0.314

Equation (7) can be employed to measure Gibbs free energy of adsorption (∆*G°*_ads_)^56^:

7 $${\Delta G}_{ads}^{0}=-RT\mathrm{ln}\left(55.5{K}_{ads}\right)$$

where *R* − 8.314 J K^−1^ mol^−1^ (universal gas constant), *T*—absolute temperature in K, and 55.5—water’s molar concentration in mol L^−1^. The negative sign of ∆*G°*_ads_ indicates that the adsorption process is spontaneous^57^.

**Figure 3:**
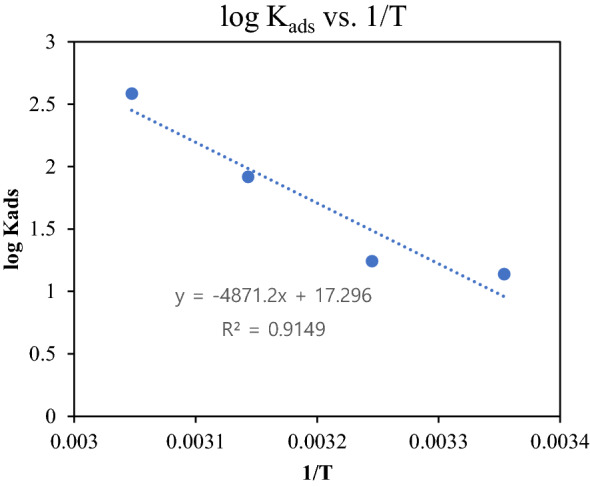
Relationship between log K_ads_ vs. 1/T.

Adsorption enthalpy change (Δ*H*^o^_ads_) was obtained by plotting to log *K*_ads_ against 1/T (Fig. 3) using Eq. (8)^57^:

8 $$\mathrm{log}{K}_{ads}=\left(-\frac{\Delta {H}_{ads}^{0}}{2.303RT}\right)+constant$$

The standard activation entropy (Δ*S*°_ads_) can be measured utilizing Vant’t Hoff Eq. (9)^58^:

9 $$\Delta {G}_{ads}^{0}= \Delta {H}_{ads}^{0}-{T\Delta S}_{ads}^{0}$$

Table 2 shows the values of Δ*S*^°^_ads_ and Δ*H*^°^_ads_ measured from Fig. 3. The positive Δ*H*^°^_ads_ values depict the endothermic nature of the activated step of the corrosion process. Instead, the negative Δ*S*^°^_ads_ values suggest that the entropy is reduces by the adherence of inhibitor molecules to a metal surface reduces^59,60^.

### Activation parameters

The increasing temperature can significantly affect a material's various properties, such as its kinetics, corrosion rate, and equilibrium. These parameters were attained through Arrhenius and transition-state Eqs. (10) and (11)^41^:

10 $$\mathrm{log}\nu =logA-\frac{Ea}{2.303RT }$$

where the pre-exponential factor is denoted by *A*, absolute temperature is indicated by *T* in Kelvin, and the Gas Constant denoted by *R* in J K^−1^ mol^−1^.

11 $$\nu = \frac{RT}{Nh}\mathrm{exp}\frac{\Delta S}{R}\mathrm{exp}\frac{-\Delta H}{RT}$$

where *N* is Avogadro’s number, and *h* is Planck’s constant.

**Figure 4:**
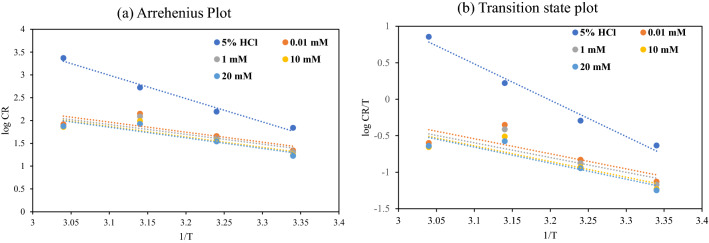
(**a**) Arrhenius plot, (**b**) transition state plot.

The activation energy of a process is computed from the log CR vs. 1/*T* plot (Fig. 4a). The lower activation energy of a process in the occurrence of a drug compared to that of a process in its absence is due to its chemical adsorption^61^. The increase in the %*η* with temperature increase also suggests chemical adsorption^62,63^.

The entropy and enthalpy of activation were computed by taking into account the Transition State (Eq. 9). From the intercept of Fig. 4b and the slope of (− ∆H*/2.303*R*), the values of ∆S* and ∆H* were determined. The ∆H* value with positive sign indicates the endothermic process of the MS, which suggests that the solution containing the synthesized inhibitor has difficulty in preventing the steel from corrosion. The reduction in the ΔS* values (Table 3) when using inhibitor-protected acid solutions suggests that the transition state is formed through an associative mechanism^64^.

**Table 3:** Activation parameters of MS in 5% HCl.

C (mM)	*E*_a_ (KJ mol^−1^)	ΔH* (KJ mol^−1^)	ΔS* (KJ mol^−1^ K^−1^)
0	97.98	95.32	0.164
0.01	42.23	39.58	− 0.027
1	41.31	38.65	− 0.031
10	43.79	41.13	− 0.024
20	44.76	42.11	− 0.022

### EIS measurement

**Figure 5:**
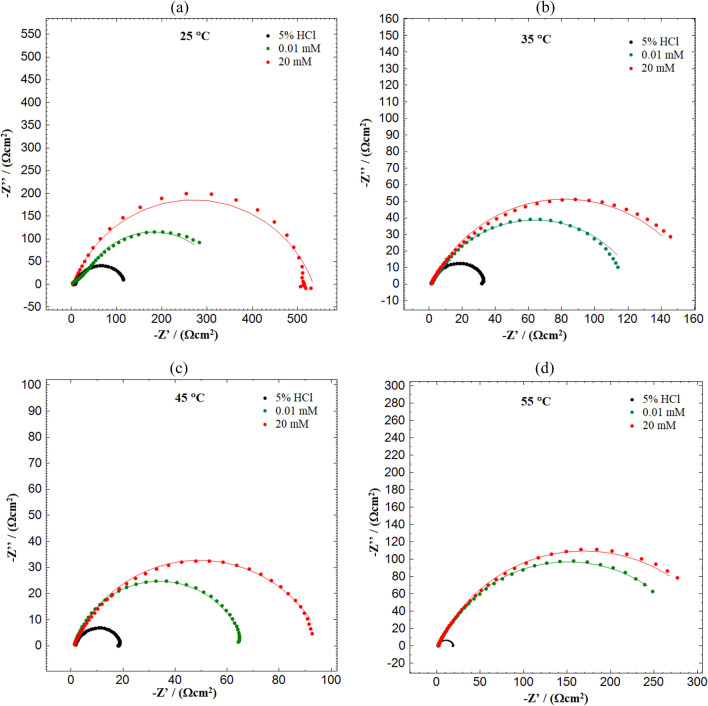
Nyquist plots for MS in 5% HCl solution in the absence and presence of different concentration of drug at (**a**) 25 °C, (**b**) 35 °C, (**c**) 45 °C, and (**d**) 55 °C.

The impact of temperature on the ampicillin drug's efficacy in 5% HCl solution containing MS was investigated at a temperature ranging from 25 to 55 °C. The electrochemical parameters namely, the polarization resistance (*R*_P_), double layer capacitance (*C*_dl_), and percent inhibition efficiencies (%η_E_), were analysed and given in Table 4. Table 4 shows that the absence and presence of the inhibitor significantly affects the these parameters. The radius of the MS capacitive loops in an inhibited solution is larger when compared to the solution with no inhibitor. As the concentration of inhibitor increases, this effect continues. The increase in the temperature leads to a decrease in the radius of the capacitive loop, but the increase in the presence of an inhibitor increases its diameter. The increase in impedance of mild steel due to the increased surface coverage of inhibitor molecules on the surface of electrode results in an increase in inhibition efficacy^65^. Additionally, Nyquist plots (Fig. 5) show a single semicircle loop for each concentration that was depressed in the centre of the plot. The capacitive loop's shape is imperfect as a result of surface imperfections and defects. Since the semicircle curves were imperfect, the constant phase element (CPE) was utilized instead of an ideal capacitor. The CPE can be calculated using the equation below^54^:

12 $${\mathrm{Z}}_{\mathrm{CPE}}= {\mathrm{Y}}_{0}^{-1}(\mathrm{j\omega }{)}^{-\mathrm{n}}$$

where *Z*_CPE_ represents the CPE’ impedance, j—an imaginary number associating to the square root of − 1, *Y*_0_—proportionality integral, *ω*—angular frequency, and *n*—the CPE exponent which is detailing about the degree of surface inhomogeneity.

**Table 4:** Electrochemical impedance characteristics for MS at different temperatures in 5% HCl solution in the absence and presence of varying concentrations of expired Ampicillin drug.

C (mM)	R_s_ (Ω cm^−2^)	R_p_ (Ω cm^−2^)	n	C_dl_ (μF cm^−2^)	%η_E_
25 °C					
0	7.4 ± 0.03	112.3 ± 0.4	0.9514 ± 0.001	206	–
0.01	12.2 ± 0.02	353.3 ± 1.1	0.9704 ± 0.002	154	68.0
20	6.5 ± 0.01	517.8 ± 2.2	0.9872 ± 0.003	122	78.2
35 °C					
0	0.94 ± 0.03	35.42 ± 3.2	0.9364 ± 0.001	725	–
0.01	2.34 ± 0.01	117.1 ± 1.8	0.9507 ± 0.003	118	69.7
20	0.48 ± 0.03	170.79 ± 3.2	0.9625 ± 0.001	76	79.3
45 °C					
0	1.62 ± 0.01	19.55 ± 1.8	0.9114 ± 0.003	863	–
0.01	0.94 ± 0.03	74.48 ± 3.2	0.9699 ± 0.001	93	73.7
20	1.62 ± 0.01	102.5 ± 1.8	0.9714 ± 0.003	81	80.9
55 °C					
0	1.58 ± 0.01	17.12 ± 0.5	0.8907 ± 0.001	978	–
0.01	0.94 ± 0.03	316.78 ± 3.2	0.9799 ± 0.001	64	94.6
20	1.62 ± 0.01	343.6 ± 1.8	0.9814 ± 0.003	13	95.0

“ ± ” shows the standard deviation of three measurements.

**Figure 6:**
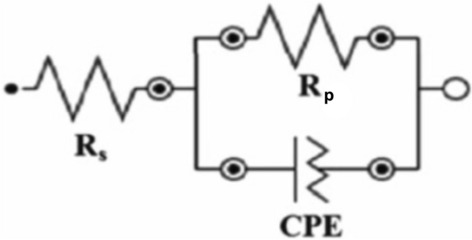
Equivalent circuit model adapted to fit the impedance data.

The data collected during the experiment were then fitted to an equivalent circuit model as illustrated in Fig. 6, which comprises the R_p_, *R*_s_ (solution resistance) and* C*_dl_. The values of *C*_dl_ were then obtained through the given equation^66^:

13 $${\mathrm{C}}_{\mathrm{dl}}={\mathrm{Y}}_{0}{({\upomega }_{\mathrm{max}})}^{(\mathrm{n}-1)}$$

where *ω*_max_ stands for angular frequency for the mythic proportion of impedance being maximum.

The data indicated that the R_p_ values escalated with the increase in the solution temperature and the concentration of the inhibitor^67,68^. The reduced *C*_dl_ values could be used to support the idea of the metal surface's ability to absorb the inhibitor molecules.

### Potentiodynamic polarization (PDP) measurement

**Figure 7:**
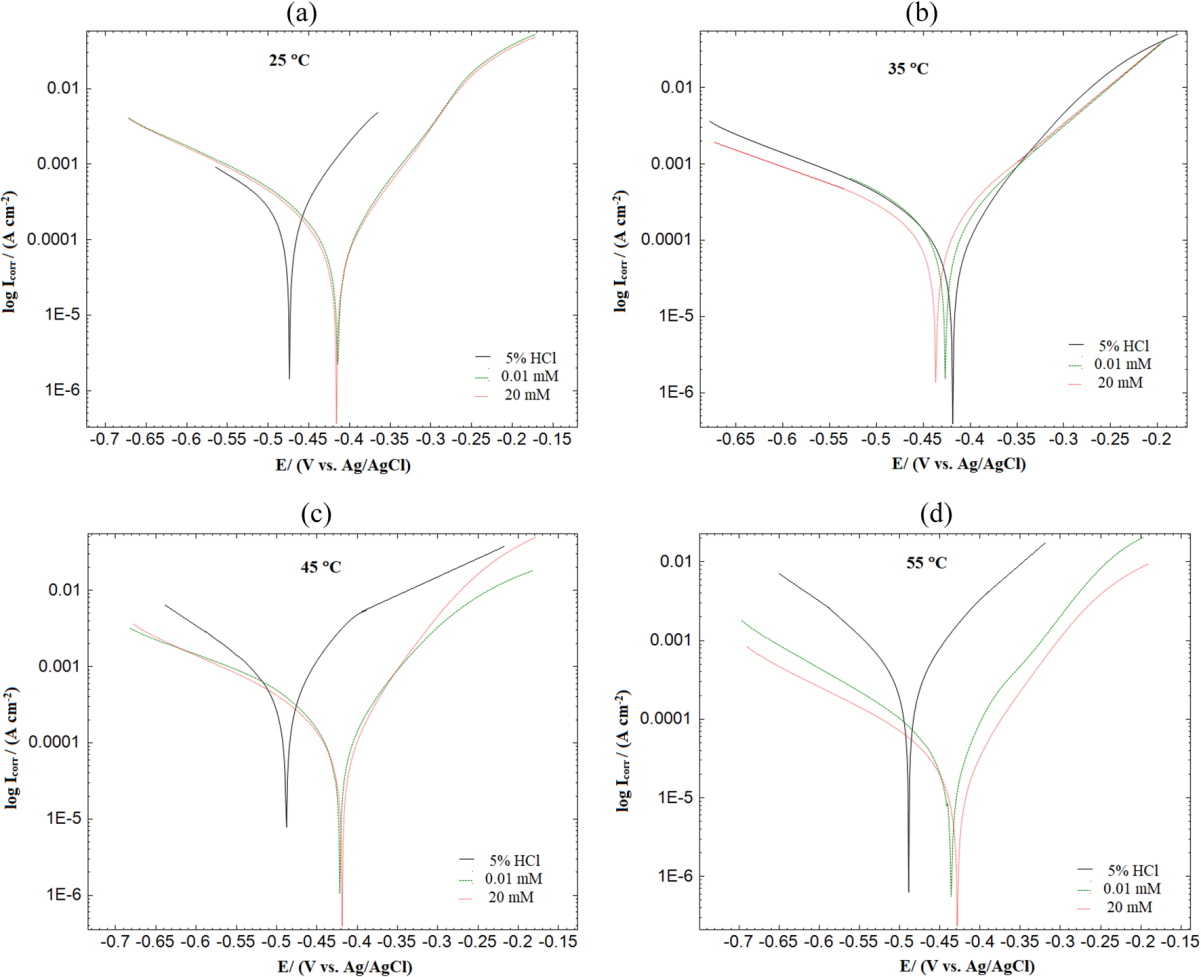
Polarization curves for MS in 5% HCl solution in the absence and presence of different concentration of drug at (**a**) 25 °C, (**b**) 35 °C, (**c**) 45 °C, (**d**) 55 °C.

Potentiodynamic polarization investigations were achieved in the temperature range of 25–55 °C in the absence and presence of 0.1, and 20 mM of expired ampicillin drug and illustrated in Fig. 7. Table 5 records the parameters extracted from PDP measurements such as *I*_corr_, corrosion potential (*E*_corr_), and %*η*_I_ of MS with and without inhibitor. It was discovered that the *E*_corr_ values of the used inhibitor differ from those of the blank solution, but the change does not exceed 85 mV, proving that these inhibitors are mixed type and reduces the values of both anodic and cathodic Tafel slope^69^. The polarization curves remain constant even when the diverse concentrations of the inhibitor are used at different temperatures (Fig. 7). The curve's shift for a lower current density indicates that the molecules' adsorption on the metal surface slowed down the corrosion process. In addition, the inclusion of an inhibitor to the blank solution results in a reduction in the values of *I*_corr_. In the absence of the studied inhibitor, *I*_corr_ was 286.2 (µA cm^−2^), which increased to reach 71.2 (µA cm^−2^) at 25 °C in the occurrence of 20 mM Ampicillin drug suggesting the adsorption of drug molecules on the metal surface. The results of the polarization curve verified that the increasing temperature caused the increase in the current density and the intensity of inhibition efficiencies. In addition, the time lag amid the desorption and adsorption of the inhibitor molecules on the metal surface increases with the temperature. The increase in the concentration and solution temperature of the inhibitor and its proficiency is consistent with the outcomes of the weight loss and the electrochemical impedance measurements. It could be due to the strong adsorption of drug molecules on the surface of the metal, which could improve the stability of the protective film of the inhibitor^70^.

**Table 5:** Potentiodynamic polarization parameters for MS at diverse temperatures in 5% HCl solution in the absence and presence of varying concentrations of expired Ampicillin drug.

*C* (mM)	*E*_corr_ (mV)	*β*_a_ (mV dec^−1^)	*β*c (mV dec^−1^)	*I*_corr_ (μA cm^−2^)	%*η*_I_
25 °C					
0	− 474.0 ± 0.01	171.6 ± 0.92	86.3 ± 0.87	286.2 ± 4.6	–
0.01	− 415.1 ± 0.01	101.8 ± 0.91	77.7 ± 0.65	89.3 ± 3.2	68.8
20	− 416.3 ± 0.02	88.7 ± 0.90	74.2 ± 0.98	71.2 ± 6.3	75.1
35 °C					
0	− 456.7 ± 0.02	129.9 ± 0.83	98.2 ± 1.1	478.4 ± 1.2	–
0.01	− 439.6 ± 0.03	125.0 ± 0.78	85.1 ± 1.5	134.1 ± 4.5	71.9
20	− 435.1 ± 0.11	125.3 ± 0.56	74.6 ± 0.21	111 ± 1.1	76.7
45 °C					
0	− 483.6 ± 0.21	125.4 ± 0.21	81.9 ± 0.23	495.6 ± 2.1	–
0.01	− 422.1 ± 0.34	118.8 ± 0.15	79.4 ± 0.45	117.1 ± 1.0	76.3
20	− 414.6 ± 0.21	99.7 ± 0.32	64.8 ± 0.67	80.2 ± 1.4	83.8
55 °C					
0	− 480.8 ± 0.54	117.9 ± 0.11	93.8 ± 0.43	378.1 ± 1.6	–
0.01	− 435.8 ± 0.21	81.2 ± 0.22	53.9 ± 0.21	21.4 ± 0.54	94.3
20	− 428.3 ± 0.11	100.5 ± 0.24	70.6 ± 0.10	16.9 ± 0.67	95.5

“ ± ” shows the standard deviation of three measurements.

### Comparative study

The results of a comparison study of the corrosion inhibition behaviour of previously reported drugs that were tested as corrosion inhibitors for various metals and media are shown in Table 6^34,71–77^. It is obvious from comparing the results that the expired drug under study has significantly more potency than other drugs. This approach improves the applicability of using the investigational inhibitor. Additionally, the fact that this inhibitor is used in low concentrations compared to previously reported drugs, can be used to explain the cost effectiveness.

**Table 6:** Comparative data showing the performance of the other drugs.

S. no.	Drug name	Biological nature of drug	Structure	Material and corrosive medium	Characterization technique	Findings	ν and %*η*	References
1	Cefdinir	Antibiotic		MS/1 M HCl,308–338 K	WL, EIS, PDP, AFM, DFT, MD	Mixed type/Langmuir adsorption isotherm	%*η*-96.9% at 6.32 × 10^–4^ M	^71^
2	Pyrazinamide	Antimycobacterial		MS/0.5 M HCl, 303–333 K	WL, PDP,EIS, SEM, DFT, MD	Mixed type/Langmuir adsorption isotherm, IE increased with temp	ν_0_-8.36 (mmpy) ν_i_-1.10 (mmpy) %*η-* 86.8%	^72^
3	Isoniazid	Antibiotic					ν_i_-0.28 (mmpy) %*η*-96.6% at 303 K ν_i_-2.86 (mmpy) %*η*-92.5% at 313 K at 1000 ppm	^72^
4	Omeprazole	Proton pump inhibitors		C38 Steel/1.0 MH_3_PO_4_	WL, PDP, EIS, SEM–EDS	Mixed type/Langmuir adsorption isotherm	ν_0_-5.99 (mg cm^−2^ h^−1^) ν_i_-0.34 (mg cm^−2^ h^−1^) IE-94.3% at 7 × 10^–4^ M	^73^
5	Ibuprofen	Anti-inflammatory, analgesic and antipyretic drug		Cu/synthetic acid rain solution	EIS, PDP, WL, QCP	Mixed (cathodic) type/Langmuir adsorption isotherm	%*η*-91% at 1 × 10^–2^ M	^74^
6	Gentamicin	Antibiotic		Al/1 M HCl	WL, HE, galvanostaticpolarization, EIS	Mixed type/Langmuir adsorption isotherm	ν_0_-0.443 (mg cm^−2^ min^−1^) ν_i_-0.048 (mg cm^−2^ min^−1^) IE-89.1% at 1000 ppm	^75^
7	Kanamycin	Antibiotic		Al/1 M HCl	WL, HE, galvanostatic polarization, EIS	Mixed type/Langmuir adsorption isotherm	ν_i_-0.039 (mg cm^−2^ min^−1^) %*η*-91.9% at 1000 ppm	^34^
8	Amikacin	Antibiotic		Al/1 M HCl	WL, HE, galvanostatic polarization, EIS	Mixed type/Langmuir adsorption isotherm	ν_i_-0.028 (mg cm^−2^ min^−1^) %*η*-91.9% at 1000 ppm	^76^
9	Seroquel	Antipsychotics		Zn/0.1 M HCl, 303–313 K	WL, EIS, PDP	Mixed type/Temkin adsorption isotherm	ν_0_- 0.010 (g cm^2^ h^−1^) ν_i_-0.0042 (g cm^2^ h^−1^) %*η*- 60.3% at 1000 ppm	^34^
10	Ciprofloxacin	Antibacterial		Bronze/simulating acid rain (pH 4)	PDP, EIS		%*η*-56.6% at 2000 ppm	^34^
11	Desloratidine	Antihistamines		CS X52/1 M HCl	WL, PDP, EIS, SEM, AFM	Cathodic type/Langmuir adsorption isotherm	%*η*–85.2% at 19.3 × 10^–5^ M at 298 K	^77^
12	Ciprofloxacin and amoxicillin	(Antibacterial		Bronze in a solution simulating acid rain (pH 4)	Polarization curves, EIS, PDP, SEM AND XPS measurements		Ciprofloxacin (2000 ppm)− 56.6%, Amoxicillin (800 ppm) − 28.8%	^34^

### Contact angle analysis

**Figure 8:**
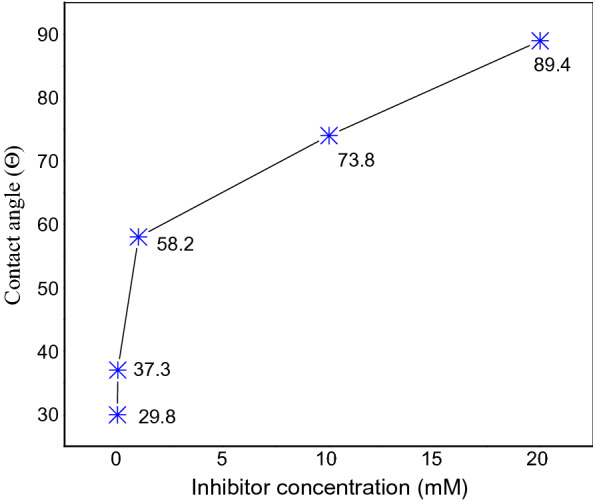
Illustrates the surface contact angles of the steel surface uninhibited with various concentrations of expired Ampicillin drug at 25 °C.

A contact angle test was executed to determine if a surface has a hydrophilic or hydrophobic characteristic. This is useful in identifying a solid substrate's ability to repel liquids. A contact angle measurement was conducted on the mild steel surface that was immersed in 5% HCl in the absence and presence of drug. The contact angle of a steel surface without an inhibitor was measured at 29.8°. The results indicated that the surface's wettability could give it favourable water-related hydrophilicity^78^. The increase in the contact angle from 29.8° to 37.3°, 58.2°, 73.8° to 89.4° with the addition of inhibitor, as shown in Fig. 8, proposes that the steel surface has a hydrophobic layer due to the adsorbed drug molecules.

### Surface morphology

**Figure 9:**
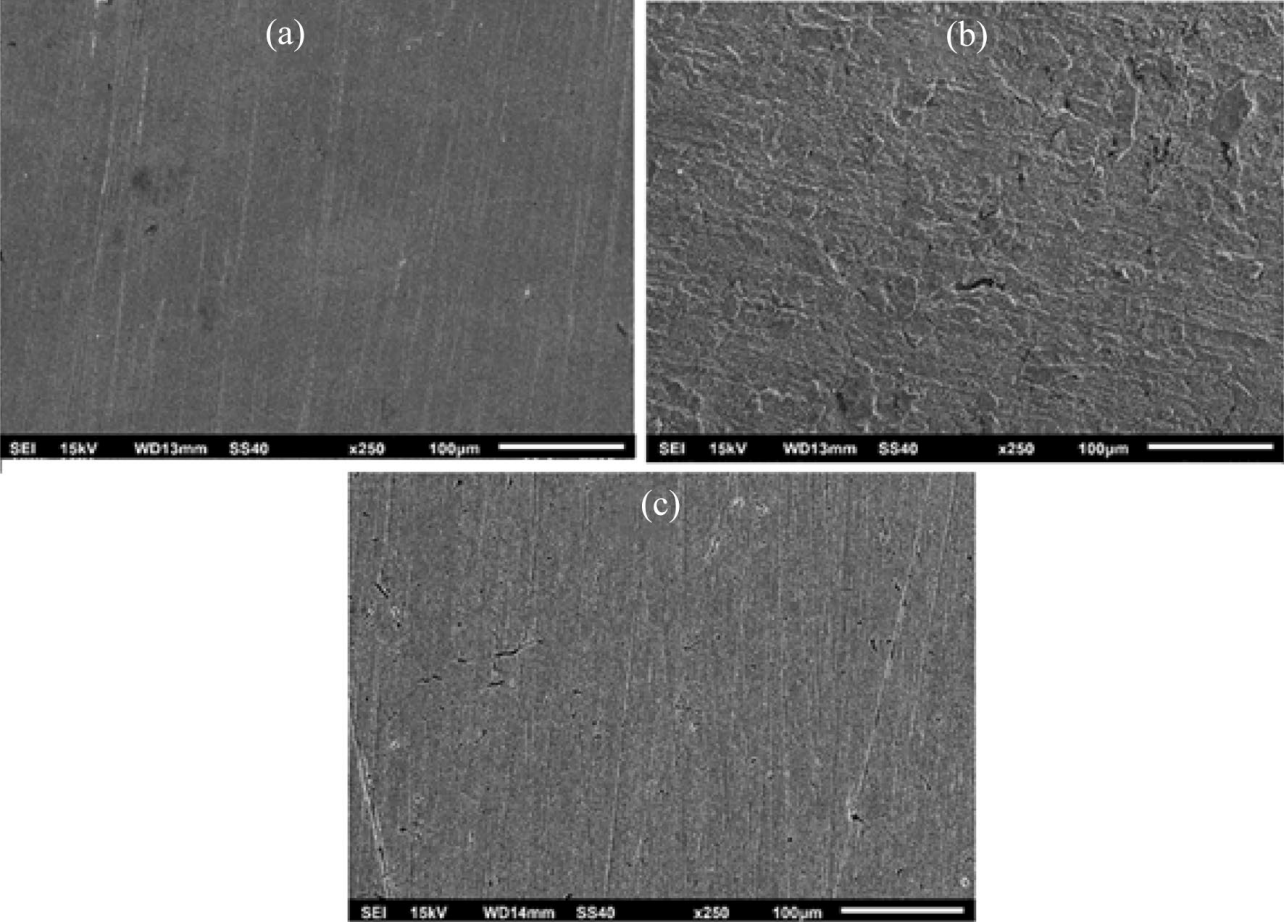
SEM micrographs of MS surface immersed in 5% HCl with (**a**) absence of inhibitor (**b**) presence of inhibitor.

The SEM micrographs were used to analyse the morphological variations in MS exhibited by 5% HCl solution. The polished MS surface exhibited a defect-free appearance Fig. 9a, while the rough MS surface was seen in the presence of 5% HCl solution because of the intense dissolution of the metal surface Fig. 9b. Figure 9c showed the changes in the surface appearance which were caused by the existence of a protective layer on the surface. This layer prevented the surface from getting rougher. The occurrence of the drug on the surface of the metal can act as a barrier against corrosion Fig. 9c.

**Figure 10:**
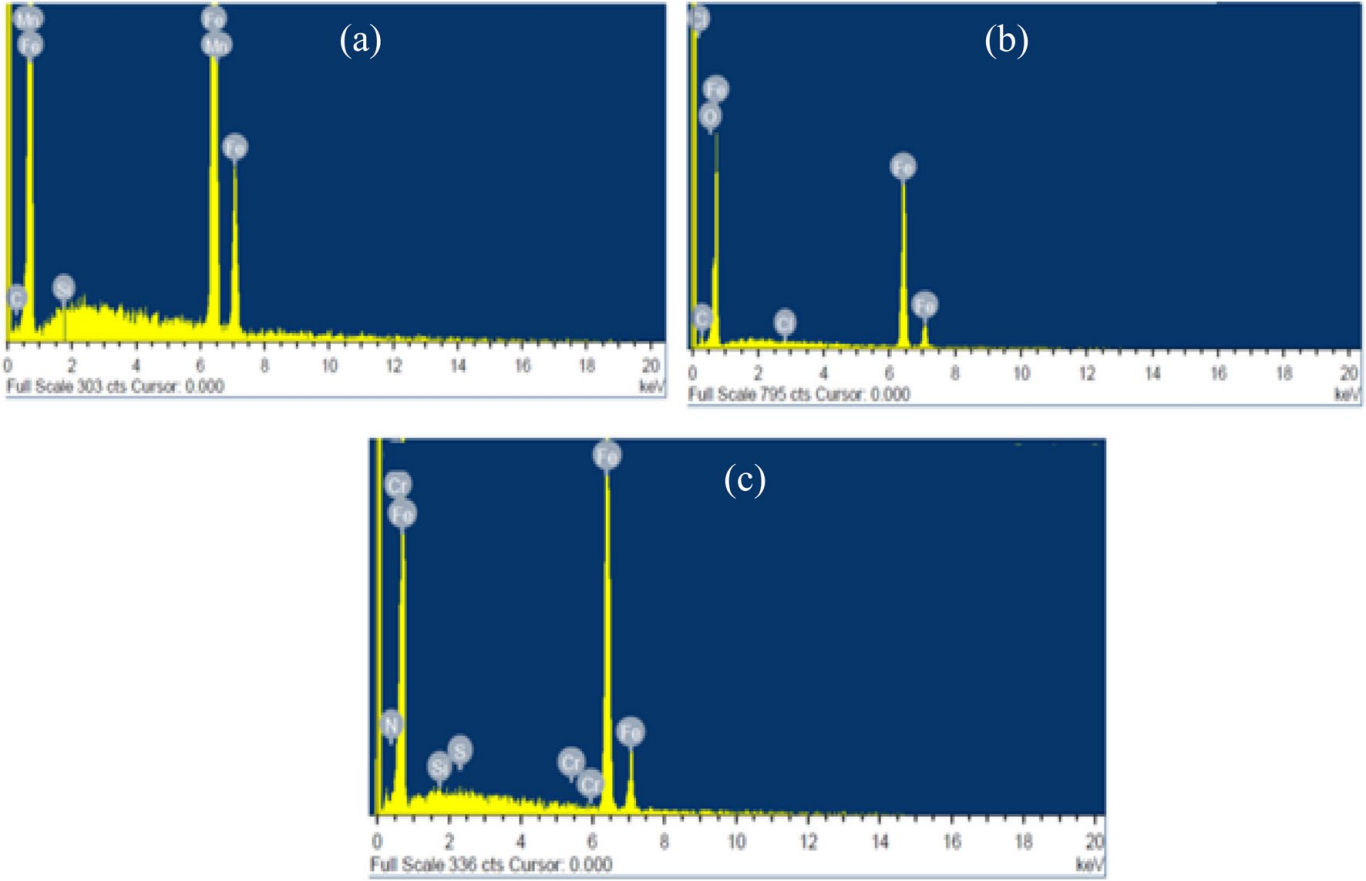
EDS photographs of MS surface immersed in 5% HCl with (**a**) absence of inhibitor (**b**) existence of inhibitor.

EDS can determine the presence of chemical composition in a sample. The surface configuration for the polished MS and MS in uninhibited and inhibited drug solutions are illustrated in Fig. 10a–c. Figure 10a displays the EDS peak of the polished MS surface before exposing it to any aggressive media, which shows some of the constituent peaks of MS. When the MS surface was exposed to 5% HCl without inhibitor (Fig. 10b), the peaks of oxygen and chlorine appeared, suggesting the formation of corrosion products in the form of oxide/chlorides due to the continuous degradation of metal in acidic phase. The absence of the oxygen peak and the increase in the peaks of iron and carbon were also observed in the 5% HCl solution. These effects led to the establishment of the surface's safety from corrosive solution because of the adsorption of inhibitor molecules (Fig. 10c).

### Mechanism of corrosion protection

In the presence of HCl solution, the MS surface is positively charged. It is well known that chloride ions have a low degree of hydration and that, through specific adsorption, they provide an excessive amount of negative charge to the metal's solution side, attracting the majority of cations^69^. In an acidic media, inhibitor molecules can remain as neutral or as protonated molecules. As a result, the adsorption modes of drug can be considered by neutral molecules or protonated forms^79^. The structure of ampicillin drug includes a benzene ring, beta-lactam ring and thiazolidine ring and heteroatoms like S, N, and O. Neutral drug molecules can be adsorbed on the mild steel’ surface via the chemisorption mechanism, which involves the water molecules’ displacement from the steel surface and the sharing of electrons between Fe and heteroatoms.

Moreover, the drug is adsorbed on the MS surface via donor–acceptor interactions among the ampicillin drug and the vacant d-orbital of iron atoms. Ampicillin heteroatoms can establish a coordinate bond by donating a lone pair of electrons to the metal's vacant d orbital. Besides that, π-electron in benzene ring may form the similar bond with the metal atom. The inhibitor molecules in the protonated form can bind to the metal surface through chloride ions, which form interconnecting bridges between the protonated drug cations and the positively charged metal surface. As a result, a densely packed layer will form on the metal surface, reducing charge and mass transfers.

## Conclusion

To develop an effective and eco-friendly corrosion inhibitor, the effect of expired ampicillin drug on the corrosion of MS in 5% HCl was studied. The study's results revealed that the drug exhibited a 96.9% inhibition efficiency at 55 °C when 10 mM of concentration of drug was used. The potentiodynamic polarization results validated that the drug exhibited mixed-type inhibition behaviour. The impedance spectra also disclosed that the inhibitor efficacy increases with the increased concentration of inhibitor, and the diameter of the semicircle increased with increased inhibitor concentration. SEM–EDS analysis confirmed the surface film formation. It can be utilized as an eco-friendly alternative to toxic corrosion inhibitors in industrial processes such as descaling and acid pickling.

## Data Availability

The data sets used and/or analysed during the current study are available from the corresponding author on reasonable request.
